# Increased temperatures reduce the vectorial capacity of *Aedes* mosquitoes for Zika virus

**DOI:** 10.1080/22221751.2019.1707125

**Published:** 2020-01-02

**Authors:** Maria Gorreti Onyango, Sean M. Bialosuknia, Anne F. Payne, Nicholas Mathias, Lili Kuo, Aurélien Vigneron, Matthew DeGennaro, Alexander T. Ciota, Laura D. Kramer

**Affiliations:** a Griffin Laboratory, New York State Department of Health, Slingerlands, NY, USA; b School of Public Health, State University of New York Albany, Albany, NY, USA; c Department of Epidemiology of Microbial Diseases, Yale School of Public Health, New Haven, CT, USA; d Department of Biological Sciences, Biomolecular Sciences Institute, Florida International University, Miami, FL, USA

**Keywords:** Climate change, *Aedes* mosquitoes, Zika virus, vectorial capacity, transmission potential

## Abstract

Rapid and significant range expansion of both Zika virus (ZIKV) and its *Aedes* vector species has resulted in ZIKV being declared a global health threat. Mean temperatures are projected to increase globally, likely resulting in alterations of the transmission potential of mosquito-borne pathogens. To understand the effect of diurnal temperature range on the vectorial capacity of *Ae. aegypti* and *Ae. albopictus* for ZIKV, longevity, blood-feeding and vector competence were assessed at two temperature regimes following feeding on infectious blood meals. Higher temperatures resulted in decreased longevity of *Ae. aegypti* [Log-rank test, χ2, df 35.66, 5, *P* < 0.001] and a decrease in blood-feeding rates of *Ae. albopictus* [Fisher's exact test, *P* < 0.001]. Temperature had a population and species-specific impact on ZIKV infection rates. Overall, *Ae. albopictus* reared at the lowest temperature regime demonstrated the highest vectorial capacity (0.53) and the highest transmission efficiency (57%). Increased temperature decreased vectorial capacity across groups yet more significant effects were measured with *Ae. aegypti* relative to *Ae. albopictus*. The results of this study suggest that future increases in temperature in the Americas could significantly impact vector competence, blood-feeding and longevity, and potentially decrease the overall vectorial capacity of *Aedes* mosquitoes in the Americas.

## Background

Zika virus (ZIKV; *Flavivirus*, *Flaviviridae*), which prior to 2007 was geographically limited to Africa and Asia [1–3], has undergone an unprecedented range expansion in recent years and evolved into a global health threat. ZIKV was first identified in Brazil in May 2015 and subsequently spread throughout the Americas [3–7]. The explosive spread of ZIKV from Asia to the Americas and the association with disease outcomes such as microcephaly among infants and Guillain-Barre syndrome among adults necessitates the need to better understand the transmission dynamics of the virus and the potential impact of temperature increases on ZIKV transmission.

Increases in average and maximum temperatures have been recorded for over a century in the contiguous United States, with an annual temperature increase of 1.7°C [8]. These increases are expected to continue to accelerate, with an additional rise of 1.6–6.6°C in the late twenty-first century [9].

It is expected that the global increase in temperature [10,11] will lead to a geographical expansion of tropical disease, particularly vector-borne pathogens, throughout temperate regions [12]. Higher temperatures are known to accelerate biochemical reactions, yet heightened metabolism can come at a cost. While temperature increases might increase viral replication, decrease the length of extrinsic incubation and accelerate development rates, there could also be a negative impact on vector survival and alterations in host-seeking behaviour that could decrease their capacity to transmit pathogens [13–16]. As an ectothermic organism, the physiology [17,18], life history [19,20] and vectorial capacity [21–23] of mosquitoes will depend significantly on changes in temperature. Successful pathogen transmission depends on mosquito susceptibility, survivability during the extrinsic incubation period (EIP), and subsequent host feeding and transmission. Hence, the impact of varying temperature on survival, feeding behaviour, vector competence and EIP differentially affects the transmission of pathogens by mosquitoes [24]. In addition, the infection rates of mosquitoes with pathogens may be dependent on the interaction between temperature and mosquito genotype [25].

Multiple studies have also demonstrated that the geographic origin of both the mosquito and the viral strain affect the vector competence of *Aedes* mosquitoes for ZIKV [1,7,26,27].

A recent study by [2] showed that a mutation in the non-structural protein 1 (NS1) may have enhanced the infectivity of ZIKV and facilitated transmission by *Aedes aegypti* in the Americas.

Comparing mosquito strains, a study by [28] reared populations of *Ae. aegypti* and *Aedes albopictus* derived from different ecological and climatic conditions under a single temperature and demonstrated high infection rates but low transmission of ZIKV for all populations. While a study by [29] reared *Ae. aegypti* populations at a different temperature from the temperature they were held during the extrinsic incubation period. Their results indicated inconsistent differences across treatments for both infection and dissemination rates of ZIKV, yet in nature, it is unlikely that there would be a sudden abrupt change in temperature from immature development to adult blood feeding.

Despite progress made to understand the biology of ZIKV infection and its interactions with its vectors, questions remain regarding the intrinsic and extrinsic factors that govern the vectorial capacity for ZIKV.

Our study aimed to define differences in vectorial capacity of unique populations of *Ae. aegypti* and *Ae. albopictus* for ZIKV held under diurnal, fluctuating temperature regimes mimicking field conditions during peak transmission in regions from which the populations were derived. Given that the Intergovernmental Panel on Climate Change (IPCC) has predicted a 2–4°C mean global temperature rise over the next century due to global warming [30], we then modelled a 2°C increase over these baseline temperatures and assessed population and species-specific differences in the effect of rising temperatures on vectorial capacity. Insights into the impact of future increase in temperature in the Americas on ZIKV transmission dynamics of *Ae. aegypti* and *Ae. albopictus* will improve our understanding of potential influence of climate change on the epidemiology of this disease in the region.

## Methods

### Mosquitoes

**Figure 1.**
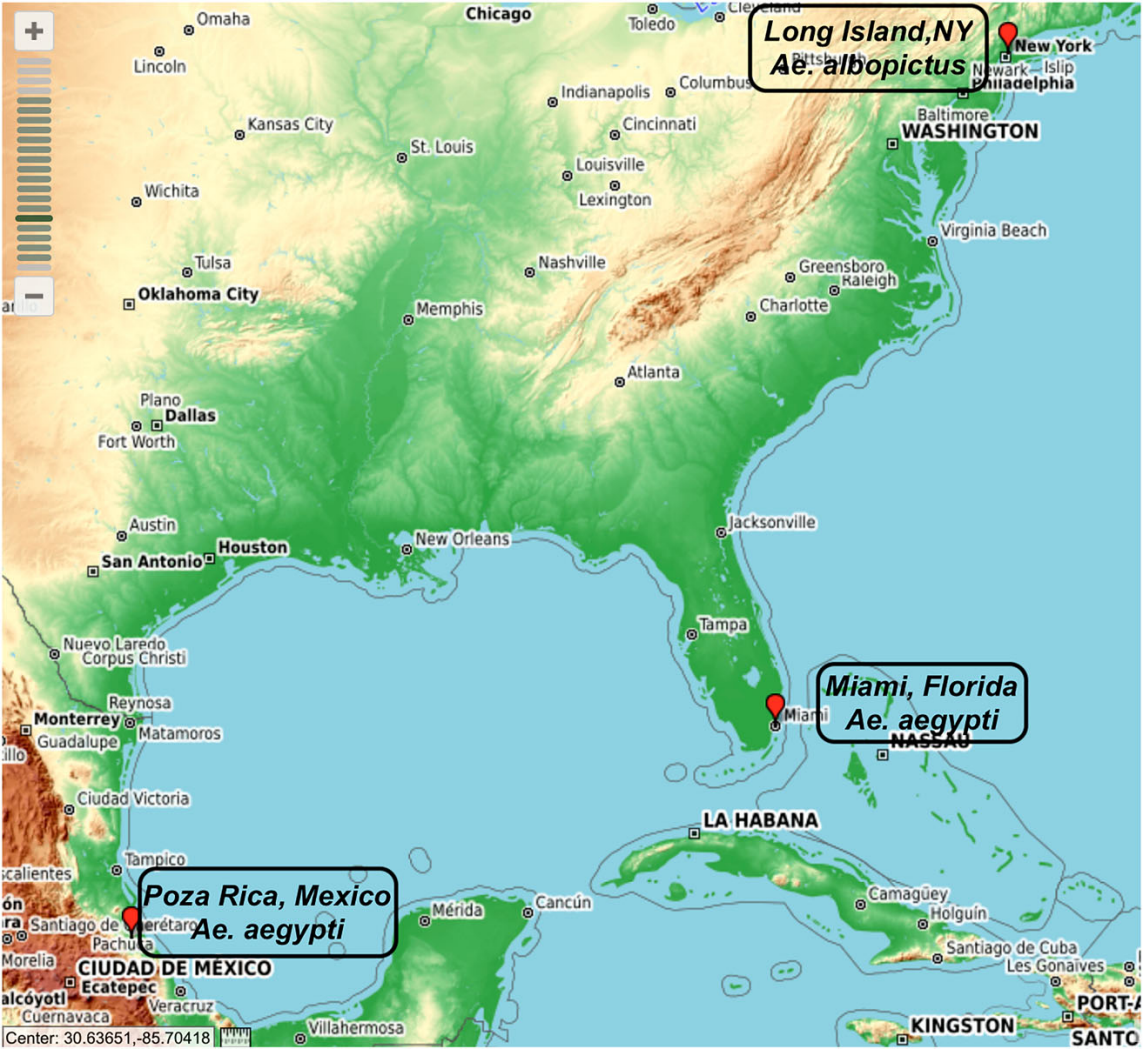
Collection sites of the *Aedes* mosquitoes. Source: https://www.gpsvisualizer.com/mapsite. Note: The AEG MI mosquitoes were collected from Miami, Florida, AEG MX population from Poza Rica, Mexico while the ALB LI from Long Island New York. The different sites of collection have different climatic conditions that include hot and humid summers, short warm winters in Miami, tropical wet and dry in Mexico and cold and temperate in Long Island, New York. The temperature used to determine the temperature regimes utilized in this study are the estimated temperatures during the peak transmission season. These include; day 30°C, night 26°C in Miami and Mexico and day 28°C, night 24°C for Long Island.

Three populations and two species of *Aedes* mosquitoes originally collected from three distinct geographical and environmental regions (Figure 1) were utilized in this study. *Ae. albopictus* (ALB LI; kindly provided by Illia Rochlin, Suffolk County Health Department) were originally collected in Suffolk County, NY in 2014 and subsequently colonized in the insectary of the New York State Department of Health (NYSDOH) Arbovirus Laboratory to F15. Mexican *Ae. aegypti* (AEG MX; kindly provided by GD Ebel, Colorado State University) were originally collected in Poza Rica, Mexico, in 2016 and maintained to F23. *Ae. aegypti* (Miami, AEG MI) were originally collected in Miami-Dade County, Florida in October 2017 (kindly provided by M DeGennaro, Florida International University) and reared to F3.

**Figure 2.**
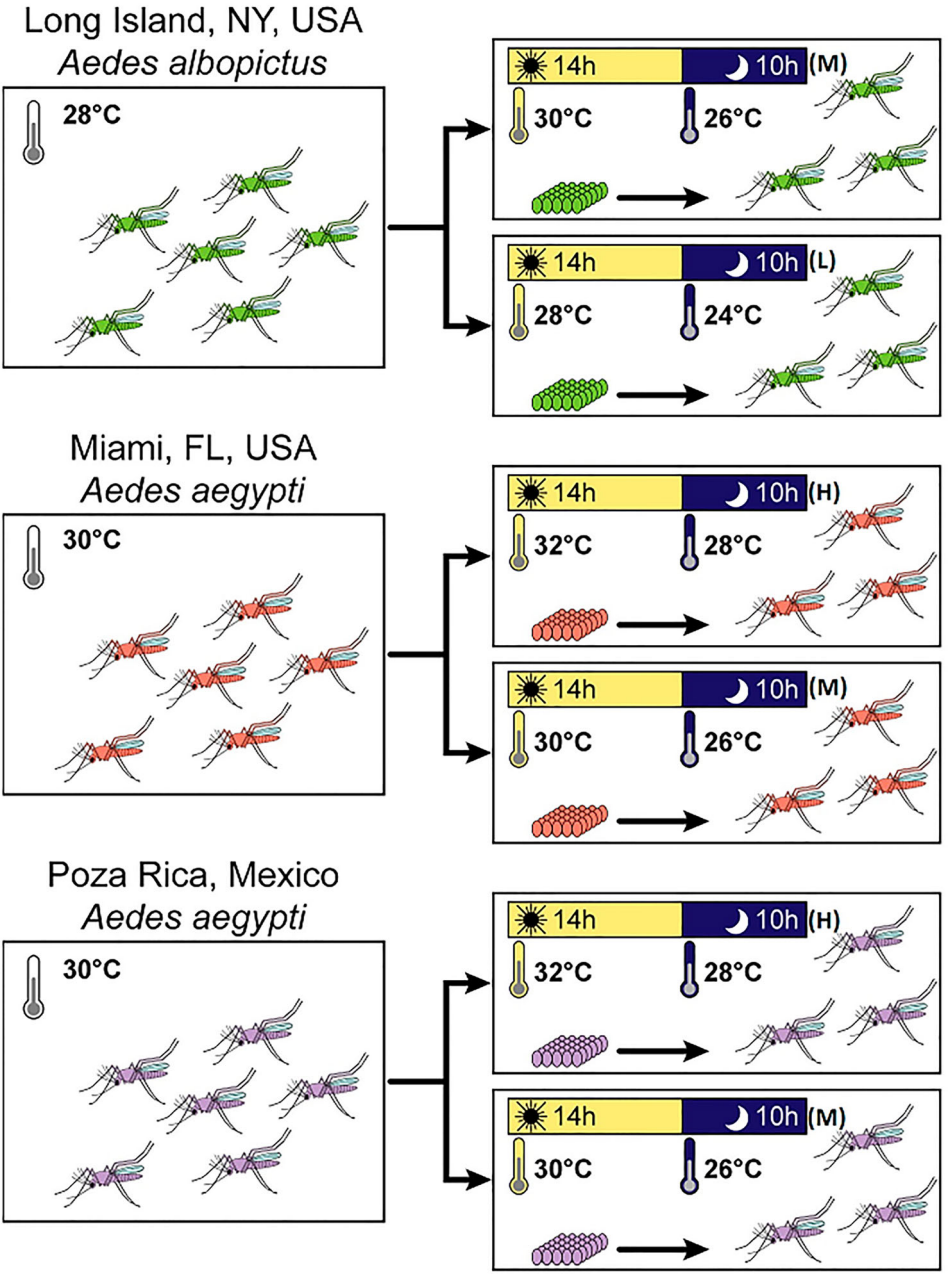
Schematic illustrating the experimental workflow. Note: The temperature regime selected for each region was based on a 2°C increase in the day and the night temperature at peak transmission season of each geographical sample origin [D30N26 (M); D32N28 (H) and D28N24 (L)]. The eggs were vacuum hatched after warming the water to the day time temperature and the immature and adult stages were reared at the stated temperature regime.

Colonies were maintained at 27°C under standard rearing conditions [14] before the eggs were hatched. F23 AEG MX, F3 AEG MI and F15 ALB LI eggs were hatched under vacuum pressure in 1 l dechlorinated water initially incubated for 3 h at the three distinct diurnal temperature regimes (Figure 2). The *Ae. aegypti* were reared and maintained at high (H) (day 32°C/ night 28°C [D32N28]) and moderate (M) (day 30°C/ night 26°C [D30N26]) temperatures; *Ae. albopictus* at M and low (L) (day 28°C/ night 24°C [D28N24]) temperature regimes. One population of *Ae. albopictus* (*Ae. albopictus* Long Island, ALB LI) and two populations of *Ae. aegypti* (*Ae. aegypti* Mexico, AEG MX and *Ae. aegypti* Miami, AEG MI) were used in this study. Baseline temperature regimes (L for *Ae. albopictus* and M for *Ae. aegypti*) were estimates of mean day and nighttime temperatures during peak transmission in regions from which populations were derived (https://www.noaa.gov). A subgroup of each population was reared and held at day and night temperatures 2°C higher than baseline (M for *Ae. albopictus* and H for *Ae. aegypti*; Figure 2).

Collected larvae were maintained in plastic rectangular flat containers [35.6 cm length × 27.9 cm width × 8.3 cm height (Sterilite, catalogue no. 1963)] at a density of 1 larva per 5 ml of dechlorinated water and reared at 40-60% relative humidity and a light–dark (LD) photoperiod 14:10 h. The larvae were fed 1.25 mg/larvae of Tetra pond Koi growth feed for first and second instar larvae and 2.5 mg/larvae for third and fourth instar larvae [27]. The larvae were counted, and all the food was pre-weighed before feeding the mosquitoes.

Both male and female adults were transferred to 3.8 l cardboard cartons upon emergence and housed together for 9 days under the different temperature regimes (Figure 2) while being provided with sugar and water *ad libitum*. To stimulate blood feeding, the 9-day old females were starved of water and sugar 18 h before an infectious blood meal. We based our duration of starvation of the mosquitoes on a standard operating procedure (SOP) for rearing mosquitoes in our insectary.

### Zika virus vector competence

To test for the effect of temperature, population and mosquito species on vector competence for ZIKV, female *Aedes* mosquitoes were orally exposed to bloodmeal containing 8.3 log_10_ PFU/ml ZIKV HND (2016-19563, GenBank accession no. KX906952) [7].

The virus was diluted 1:1 with defibrinated sheep blood plus 2.5% sucrose; sodium bicarbonate was included to adjust pH to 8.0. The female mosquitoes were offered the infectious blood meal through a 37°C pre-heated Hemotek membrane feeding system (Discovery Workshops, Acrington, UK) with a porcine sausage casing membrane. After an hour, the mosquitoes were anaesthetized with CO_2_ and immobilized on a pre-chilled tray connected to 100% CO_2_. Engorged females were separated and placed in three separate 0.6 l cardboard cartons (30 individuals per carton). In addition, 1 ml of each blood meal was transferred to a 1.5 ml Safe Seal microtube (Eppendorf, Hamburg Germany) and stored at −80°C to allow for the determination of ZIKV titres. The engorged females were maintained on 10% sucrose solution provided *ad libitum*. The 0.6 l cardboard cartons were kept at the respective temperature regimes (Figure 2).

### Infection and transmission analysis

On days 4, 7 and 14 post-infectious blood meal, mosquitoes were immobilized by exposure to triethylamine (Sigma Aldrich, St. Louis, MO, USA), the legs were removed from 30 mosquitoes of each population and species, and placed in 500 µl mosquito diluent (MD; 20% heat-inactivated foetal bovine serum in Dulbecco phosphate-buffered saline plus 50 µg/ml penicillin/streptomycin, 50 µg/ml gentamicin and 2 µg/ml Fungizone [Sigma Aldrich, St. Louis, MO, USA]) containing a 4 mm bead (Daisy Rogers, Arkansas). Saliva was collected by inserting the proboscis of the female mosquitoes into a capillary tube containing ∼20 µl foetal bovine serum plus 50% sucrose 1:1 for 30 min and subsequently ejecting the mixture into 125 µl MD. Mosquito bodies were then placed in individual tubes containing 500 µl MD and a bead. All samples were held at −80°C until assayed.

Infection, dissemination and transmission results were obtained by screening the whole bodies, legs and saliva, respectively, collected at different time points, as described by [7]. To obtain viral titre, ZIKV-specific quantitative PCR assay that targets the NS1 region was utilized [31].

### Statistical analysis

Statistical analysis was performed with GraphPad Prism version 5.0. Infection (body); dissemination (legs) and transmission (saliva) rates were compared using Fischer’s exact test. Viral loads were compared using an ANOVA test.

### Vectorial capacity

Vectorial capacity (VC), which is a calculation of the probability that an individual mosquito in a given population will transmit a pathogen, incorporates blood-feeding behaviour and vector longevity along with vector competence. In this study, VC was calculated using the following formula [32–34]:

(1) $$\mathrm{VC}=h*p^{N}*b/-\ln(p)$$

where *h* = host feeding rate (proportions of mosquitoes acquiring at least two artificial blood meals in their lifetime) and *p* = the probability of daily survival. Death of each individual was plotted in days and a linear regression analysis with a runs test was used to determine if the relationship between time and mortality is linear. The slope of the best fit line is used to calculate *p* as follows, *p* = (100 − [ − slope]). *N* = the mean extrinsic incubation period (EIP), calculated here as the mean days to the transmission and *b* = vector competence (mean proportion of exposed mosquitoes transmitting days 7–14). The host feeding rate and the longevity were evaluated in a separate study using the same populations and temperature regimes. Briefly, six populations of *Aedes* mosquitoes were reared at similar temperature regimes and conditions as described above. Eggs were vacuum hatched as described before [1360 eggs AEG MI (hatched and reared at the H temperature regime); 1360 eggs AEG MI (hatched and reared at the M temperature regime); 1047 eggs AEG MX (hatched and reared at the H temperature regime); 952 eggs AEG MX (hatched and reared at the M temperature regime); 750 eggs ALB LI (hatched and reared at the M temperature regime) and 721 eggs ALB LI (hatched and reared at the L temperature regime)], and the larvae reared as described above.

The emerged female mosquitoes were allowed to mate and after three days, then placed in separate 3.8 l cartons and offered a non-infected blood meal. Mortality was monitored and recorded daily. Blood meals were subsequently offered every three days four times, until the last female mosquito died. The linear regression analysis of these data was completed using GraphPad Prism 5.0.

## Results

### Vector competence

**Figure 3.**
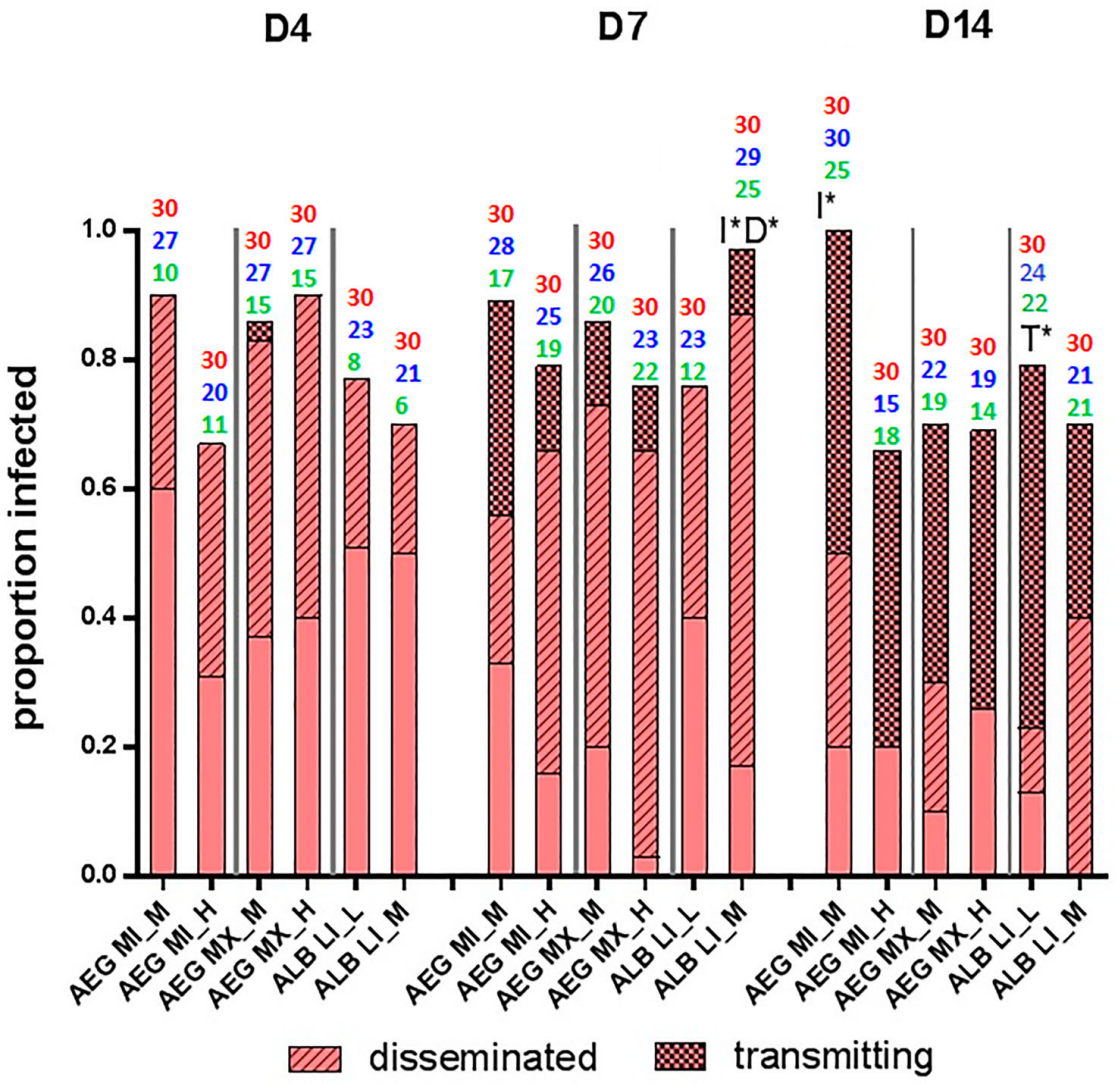
Vector competence of *Aedes* mosquitoes infected with Zika virus and reared at different temperature regimes. Note: The height of the bar plot represents the proportion of blood-fed individuals successfully infected with Zika virus. Above each bar plot, the total number of females screened for infection is indicated in red, the total number screened for dissemination, blue and transmission, green. The proportion of these that disseminated and subsequently transmitted the virus are further indicated. The temperature affected infection and dissemination rates in a population and species-specific manner. Increased temperature reduced the infection rates of AEG MI at the last time point (14 dpi) [Fisher’s exact test *P* < .05]. The opposite effect was measured in ALB LI, with significantly higher infection [Fisher’s exact test *P* < .05] and dissemination rates [Fisher’s exact test *P* < .05] with an increase in temperature at 7 dpi. The ALB LI (L) population had the highest transmission efficiency [Fisher’s exact test *P* < .05]. I* represents significantly different infection rates *P* < .05; D* represents significantly different dissemination rates *P* < .05; T* represents significantly different transmission efficiency *P* < .05, when comparing among temperature regimes.

*Ae. aegypti* and *Ae. albopictus* were equally susceptible to ZIKV infection [Fisher’s exact test *P* = .44]. Overall, temperature influenced infection rates in a population and species-specific manner. Higher temperatures were associated with lower ZIKV infection rates of AEG MI population, i.e. the individuals reared and held at baseline temperature (M) had a significantly higher proportion of individuals infected (85/90) as compared to those reared at higher temperatures (H) (67/90) [Fisher’s exact test *P* < .001]. However, the temperature did not influence infection rates of the AEG MX population [H (72/90) M (76/90) Fisher’s exact test *P* = .56]. Although the temperature did not influence overall infection rates of the ALB LI population [M (71/90) L (73/90) Fisher’s exact test *P* = .85], we noted differences at 7 dpi. Specifically, an increase in temperature significantly increased infection of ALB LI individuals [M (29/30) L (23/30) Fisher’s exact test *P* < .05] (Figure 3).

There were no significant differences in dissemination or transmission rates among temperature regimes, with the exception of the ALB LI population. At 7 dpi, increased temperature significantly increased ALB LI dissemination [M (25/29) L (12/23) Fisher’s exact test *P* < .05]. Conversely, at 14 dpi the ALB LI L population had a significantly higher transmission efficiency than the ALB LI M population [Fisher’s exact test *P* < .05] (Table 1; Figure 3).

**Table 1.** Vector competence of *Aedes* mosquitoes in this study following ZIKV exposure.

Species	Geographical origin	Temp. regime	% Infection (whole bodies)	% Dissemination (infected bodies)	% Transmission (disseminated bodies)	% Transmission efficiency (no. of mosquitoes initially screened)
4 days post-infection						
*Ae. aegypti*	Miami	D32N28 (H)	67 (30)	55 (20)	0 (11)	0 (30)
*Ae. aegypti*	Miami	D30N26 (M)	90 (30)	37 (27)	0 (10)	0 (30)
*Ae. aegypti*	Mexico	D32N28 (H)	90 (30)	56 (27)	0 (15)	0 (30)
*Ae. aegypti*	Mexico	D30N26 (M)	90 (30)	56 (27)	13 (15)	7 (30)
*Ae. albopictus*	Long Island	D30N26 (M)	70 (30)	29 (21)	0 (6)	0 (30)
*Ae. albopictus*	Long Island	D28N24 (L)	77 (30)	35 (23)	0 (8)	0 (30)
7 days post-infection						
*Ae. aegypti*	Miami	D32N28 (H)	83 (30)	76 (25)	26 (19)	17 (30)
*Ae. aegypti*	Miami	D30N26 (M)	93 (30)	61 (28)	6 (17)	3 (30)
*Ae. aegypti*	Mexico	D32N28 (H)	77 (30)	96 (23)	14 (22)	10 (30)
*Ae. aegypti*	Mexico	D30N26 (M)	87 (30)	77 (26)	21 (20)	13 (30)
*Ae. albopictus*	Long Island	D30N26 (M)	97 (30)	86 (29)	12 (25)	10 (30)
*Ae. albopictus*	Long Island	D28N24 (L)	77 (30)	52 (23)	0 (12)	0 (30)
14 days post-infection						
*Ae. aegypti*	Miami	D32N28 (H)	67 (30)	80 (15)	78 (18)	47 (30)
*Ae. aegypti*	Miami	D30N26 (M)	100 (30)	83 (30)	60 (25)	50 (30)
*Ae. aegypti*	Mexico	D32N28 (H)	70 (30)	63 (19)	93 (14)	43 (30)
*Ae. aegypti*	Mexico	D30N26 (M)	73 (30)	86 (22)	68 (19)	43 (30)
*Ae. albopictus*	Long Island	D30N26 (M)	70 (30)	100 (21)	43 (21)	30 (30)
*Ae. albopictus*	Long Island	D28N24 (L)	83 (30)	91 (24)	71 (22)	57 (30)

**Figure 4.**
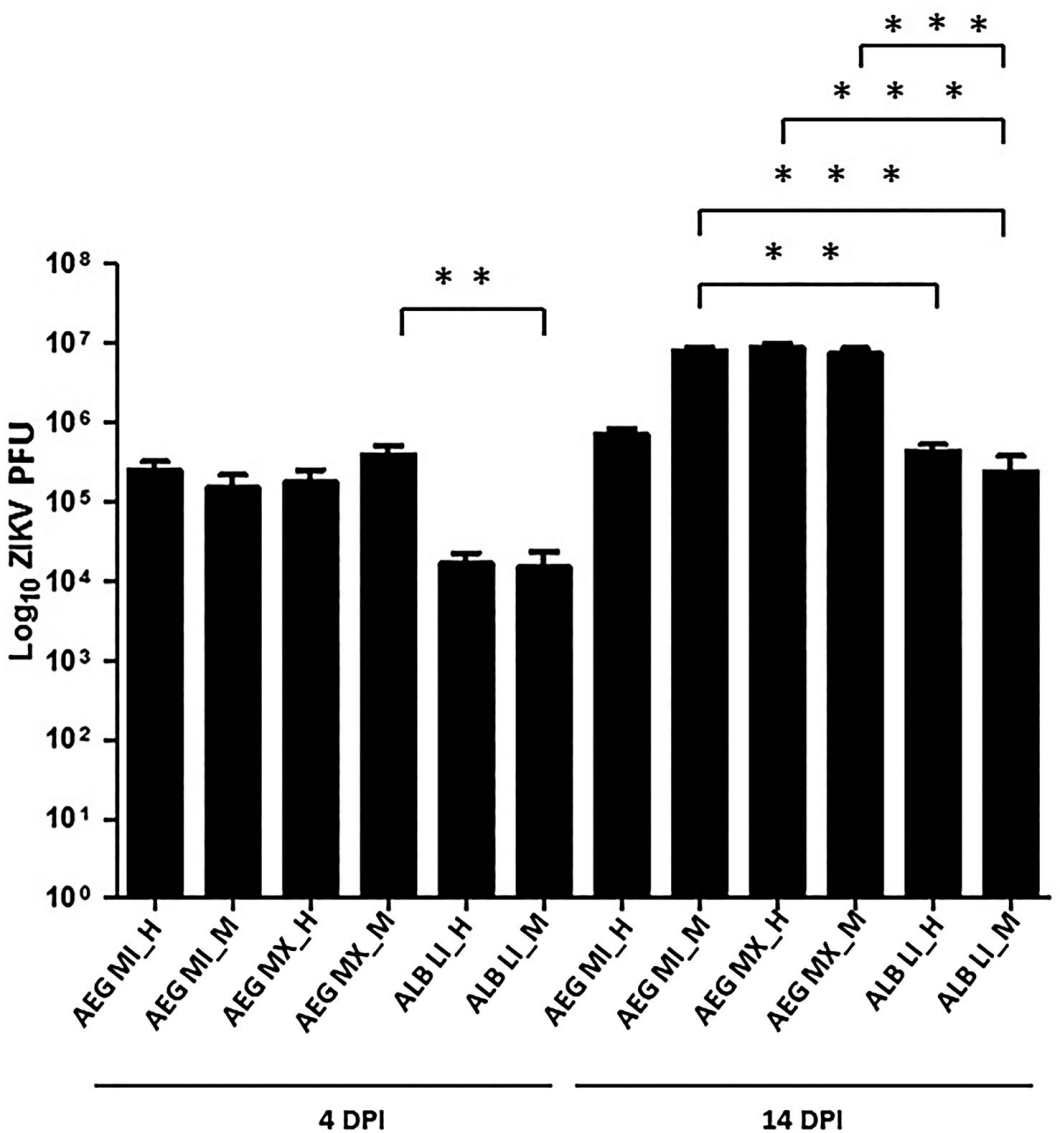
Viral load of ZIKV in *Aedes* mosquitoes at 4 and 14 days post-infection (DPI). Note: The plot represents the absolute viral load of ZIKV in individuals of different populations of *Aedes* mosquitoes utilized in this study. The distribution of the viral titre around the mean is represented by the error bar. The crossbars in the figure are indicative of a statistically significant heterogenous viral titres measured between populations. The viral load was determined by quantitative PCR for mosquitoes held at L (D28N24), M (D30N26) and H (D32N28) temperature regimes. Statistically significant differences were determined by 1-way ANOVA test indicated by *P < .05, **P < .01 and ***P < .001.

Significant differences in viral titre were measured in whole-body samples at 4 and 14 dpi (ANOVA, Tukey HSD post-hoc test, *P* < .001). At 14 dpi, a significantly higher viral load was measured in AEG populations relative to ALB LI populations (ANOVA, Tukey HSD post-hoc test, *P* < .001) (Figure 4). No significant effect of population or temperature on viral load was measured.

### Vectorial capacity

**Figure 5.**
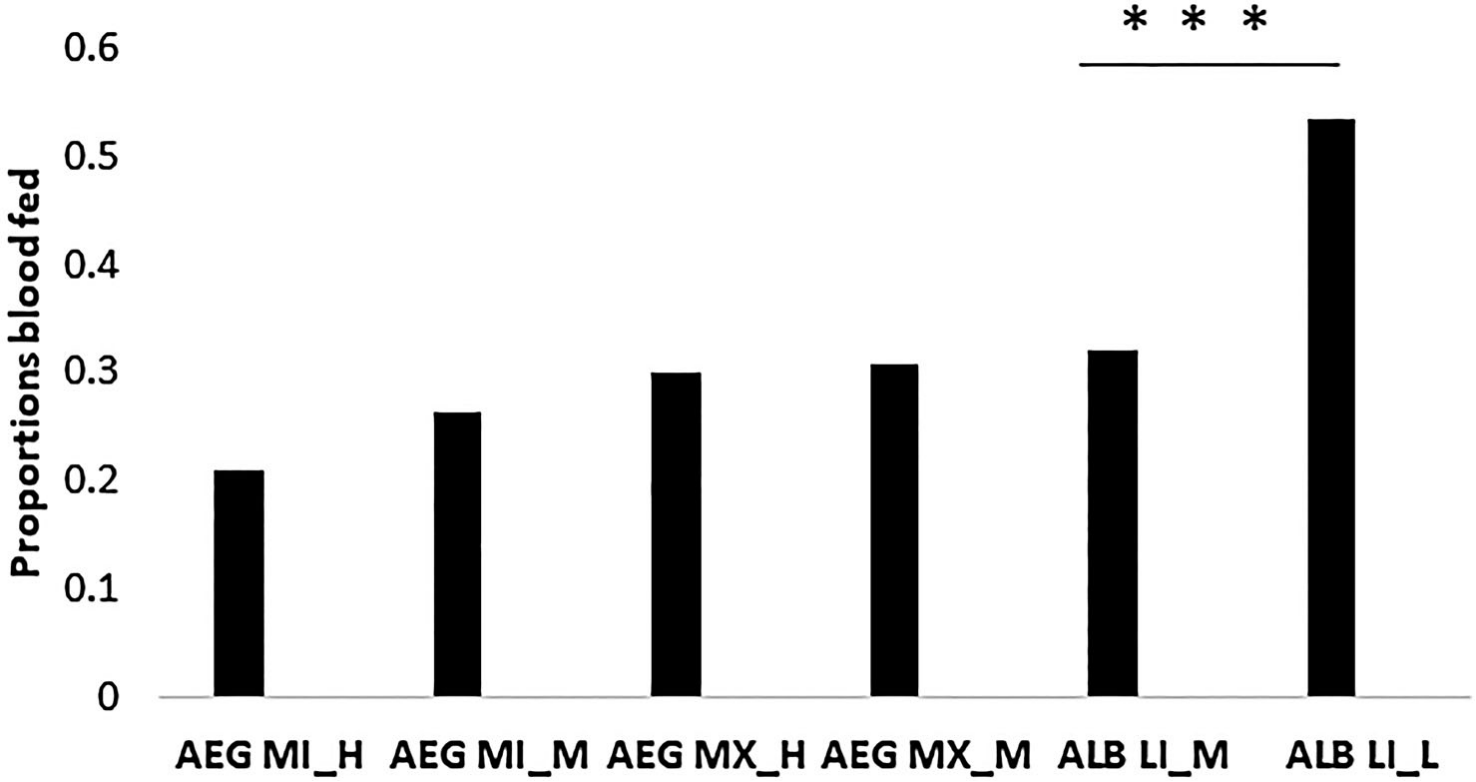
Blood feeding frequency of *Aedes* mosquitoes at different temperatures. Note: The bar graphs represent the frequency of blood meal acquisition for individuals offered non-infectious blood meals twice a week post-mating. Mosquitoes were reared at similar temperature regimes as described for vector competence experiments: including L (D28N24), M (D30N26) and H (D32N28) regimes. Significantly higher feeding rates were measured for the ALB LI population at higher temperatures ***Fisher’s exact test (*P* < .001).

**Figure 6.**
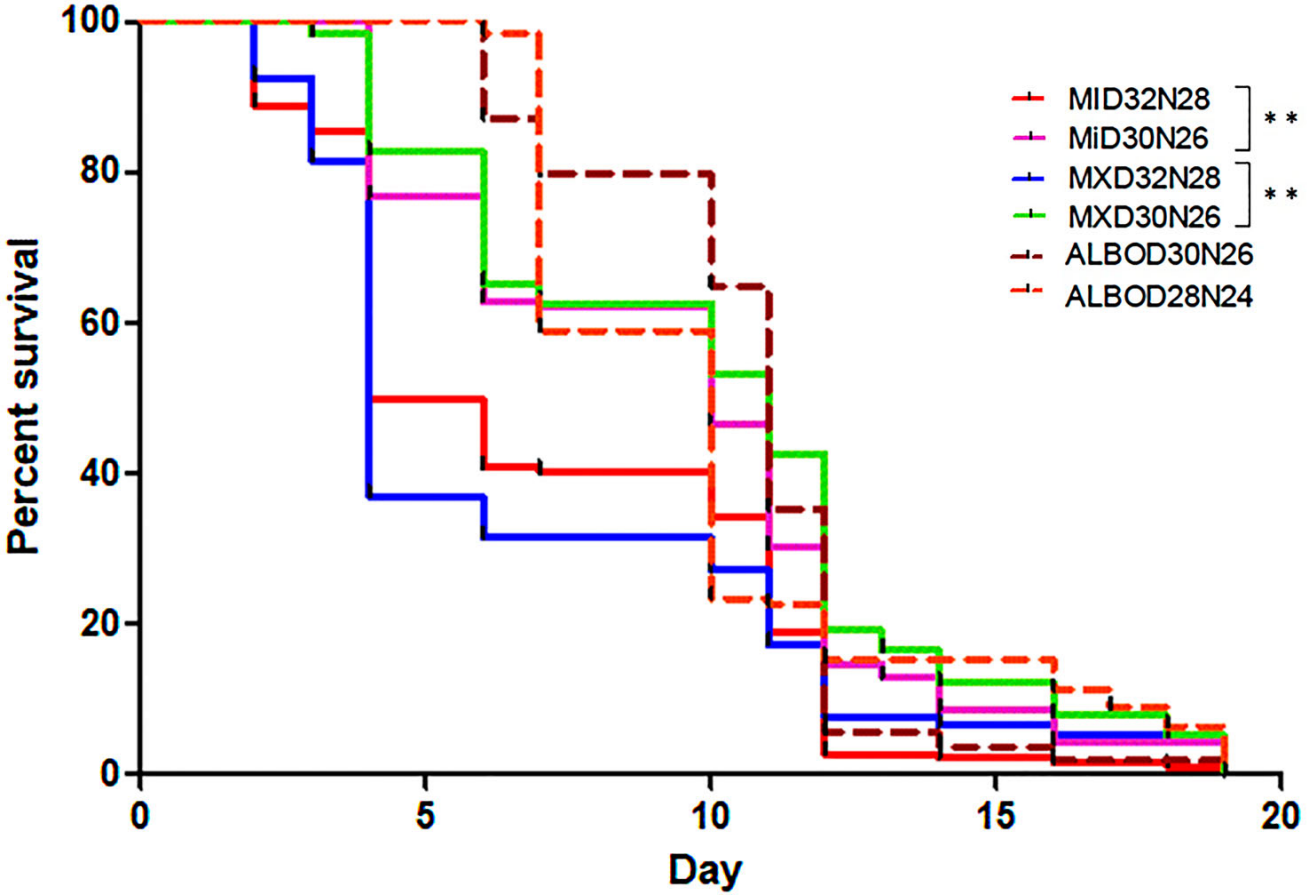
The longevity of *Aedes* mosquitoes at varying temperature regimes. Note: Mosquitoes were reared at similar temperature regimes as described for vector competence experiments: including L (D28N24), M (D30N26) and H (D32N28) regimes. Mortality was monitored and recorded daily. Graphs are Kaplan–Meier survival curves depicting proportions surviving at various days post-emergence. Significant differences were measured between populations and temperature regimes **Log-rank test (*P* < .01).

Increased temperature resulted in decreased blood feeding frequency by ALB LI population in [M (32%), L (53%) Fisher’s exact test *P* < .001] (Figure 5) while the AEG MI [M (36%), H (27%) Fisher’s exact test *P* = .09]and AEG MX [M (44%), H (43%) Fisher’s exact test *P* = .92] populations blood-feeding frequencies were not significantly affected. *Ae. aegypti* reared at the H temperature regimes had significantly reduced longevity relative to the M temperature regime population (Log-rank [Mantel–Cox] test, Chi-square, df 35.66, 5, *P* < .001; Figure 6). *Ae. albopictus* longevity was not affected by temperature (Log-rank [Mantel–Cox] test, *P* > .001).

Overall, ALB LI (L) had the highest vectorial capacity (0.53), while AEG MI (H) had the lowest (0.09). Across all populations, increasing temperatures decreased vectorial capacity (Table 2). When comparing both the *Ae. aegypti* and *Ae. albopictus* populations at the same temperature regime (M), vectorial capacity was modestly higher for *Ae. aegypti* (ALB LI (M) = 0.17, AEG MI (M) = 0.26, AEG MX (M) = 0.33; Table 2). The decreased transmissibility at a higher temperature in *Ae. albopictus* can be wholly attributed to decreased feeding frequency while decreases in *Ae. aegypti* are influenced primarily by decreased longevity, but additionally by decreased feeding, decreased competence and increased EIPs (Table 2).

**Table 2.** Vectorial capacity of the *Aedes* mosquito populations for ZIKV.

	Source	Temp. regime	*h*	*P*	*N*	*b*	VC = *h*^2^*P^N^b*/−ln(*P*)
AEG MI (H)	Miami	D32N28	0.21	.92	11.12	0.30	0.09
AEG MI (M)	Miami	D30N26	0.30	.94	8.17	0.42	0.26
AEG MX (H)	Mexico	D32N28	0.26	.92	11.38	0.27	0.08
AEG MX (M)	Mexico	D30N26	0.31	.95	8.06	0.27	0.33
ALB LI (M)	Long Island	D30N26	0.32	.94	10.50	0.20	0.17
ALB LI (L)	Long Island	D28N24	0.53	.94	14.00	0.28	0.53

## Discussion

This study demonstrates that increasing temperatures decreased the vectorial capacity of *Aedes* mosquitoes for ZIKV. Measured decreases in vectorial capacity resulted from alterations to vector competence, blood-feeding frequency and /or longevity. While the magnitude of these effects was dependent on species, population and temperature regime, an overall decrease in ZIKV transmissibility was measured with all three populations evaluated following a 2°C rise in diurnal temperature cycle. A larger decrease in ZIKV transmission was measured for *Ae. aegypti* mosquitoes when modelling warmer current and predicted future temperature regimes of the southern US and Mexico. These results support the idea that future temperature increase could result in a northern shift in the suitability of *Aedes* populations for transmission of ZIKV and other invasive arboviruses.

Higher temperatures decreased the blood-feeding rates of the *Aedes* mosquitoes in this study, although this difference was only significant for *Ae. albopictus*. *Ae. aegypti* has been shown to take several blood meals in a single gonotrophic cycle [35] and the frequency of their blood feeding has been noted to be positively correlated with the temperature of the environment [36]. However, this correlation has been attributed to indirect effects of environmental temperature on the mosquito development, energy storage and rate of blood meal digestion [36,37]. A caveat in our study is that *Ae. aegypti* populations were reared at a higher temperature of 32°C but the high temperature for *Ae. albopictus* was 30°C. While this was done to model current (and future) temperatures in locations where these populations were derived, it is not clear how further increases in temperature above 30°C would affect this population of *Ae. albopictus*. Previous studies demonstrate that population differences have a highly significant effect on the influence of temperature on feeding behaviour [27]. In spite of this, our results suggest future climate change resulting in increased temperature may lead to a reduction in vectorial capacity of *Aedes* mosquitoes.

Overall, increased temperature of 2°C negatively impacted the longevity of the *Ae. aegypti* populations while *Ae. albopictus* were not affected. The higher survival capacity of *Ae. albopictus* both in the field and laboratory settings could contribute to these differences. Since [38] showed a wider temperature tolerance of *Ae. aegypti* up to 40°C, the contribution of senescence may also play a role in the difference between our findings and [38].

At all rearing temperatures, we noted that there was a higher proportion of males compared to females on day one and two of emergence, as is generally seen. Three days after emergence, we blood fed the females and immediately separated the engorged females from the males. Studies have demonstrated that when female *Aedes* mosquitoes are associated with the males, they lived longer [39,40]. The male reproductive gland substances deposited by the male during mating increase female fitness [41]. The reason for a shorter survival time (19 days) may be due to the fact that a higher percentage of the females may not have mated before their first blood meal hence reducing their longevity.

In our study, the increased temperature did not consistently increase competence or accelerate EIP. In fact, for *Ae. aegypti*, overall competence was either higher or statistically similar at the lowest temperature regime and EIP was shorter. Our findings were contrary to the findings of [24] who demonstrated a unimodal effect of temperature on the EIP of ZIKV on *Ae. aegypti* across eight constant temperatures (16–38°C), with the shortest EIP at 36.4°C and the peak infectivity at 30.6°C. In addition to the use of unique populations and strains, a critical difference between our study and studies that have found a direct correlation between temperature and EIP [42–44] is that our mosquitoes were reared under fluctuating diurnal temperatures. Unlike adult holding temperature, some previous studies have found an indirect correlation between larval rearing temperatures and vector competence [45–47]. This effect could be related to altered RNA interference functionality at different temperatures [18]. In addition, fluctuation of temperatures has been shown to result in reduced longevity, lower midgut infection rates and increased EIP for virus compared to a constant temperature [48–51].

Viral loads in *Ae. albopictus* reared at the M temperature regime were significantly lower than *Ae. aegypti* reared at the M temperature regime across time points. Our results corroborate findings of [7] with experiments conducted at 28°C. Previous studies have demonstrated *Ae. albopictus* is a competent vector for ZIKV in the laboratory [1,7]. However, the vector competence has been shown to be influenced by the ZIKV strain and the geographic origin of the mosquito population [1]. The explosive spread of ZIKV in the Americas raised concerns that another vector in addition to *Ae. aegypti* might be involved in ZIKV transmission [52]. Our study confirms the potential for *Ae. albopictus* to play a role in ZIKV epidemics in the Americas, although they have not been demonstrated to be significantly involved to date.

However, since variation in vector competence among *Ae. aegypti* populations and virus strains has been reported previously [7,53–55], and our results further support this, a comprehensive assessment of the potential effect of increase in temperature would require additional studies with multiple populations and ZIKV strains.

There are two important caveats to this study. First, it is unclear how mosquitoes will adapt or evolve in response to incremental changes in temperature and this could significantly alter the vectorial capacity of future populations. Second, alterations to life-history traits including blood-feeding behaviour and longevity can result from arbovirus infection [56].

We measured lower infection rates at later timepoints, particularly at high temperatures. Since arboviral infections in mosquitoes are persistent, these data suggest that ZIKV infected individuals were less likely to survive the experimental period and that this increased mortality was facilitated by increased temperatures. Further studies monitoring life-history traits following infection at altered temperatures will help clarify this relationship.

Taken together, these data demonstrate that temperature is a significant component of ZIKV transmission; and defining optimum conditions for individual populations and species is important for an accurate prediction of how future increases in temperature will affect geographic distribution and intensity of transmission in the Americas.

## Conclusion

The results of our study suggest that future global climate change resulting in increased global temperature will have negative fitness cost on *Aedes aegypti* mosquitoes for ZIKV and could significantly alter their vector competence. Further, the impact of future climate change will be driven by unique intrinsic interactions between specific vectors and pathogens and therefore is unlikely to be uniform across different species and populations [24].
